# Letter to the Editor: “Self-Compassion in migraine: associations with depression, anxiety, and clinical characteristics”- methodological and statistical considerations

**DOI:** 10.1055/s-0046-1829166

**Published:** 2026-10-23

**Authors:** Etika Rana

**Affiliations:** 1 Maharishi Markandeshwar (Deemed to be University), Maharishi Markandeshwar Institute of Physiotherapy and Rehabilitation, Mullana, HaryanaIndia.

Dear Editor,

The recently published article, *“* Self-compassion in migraine: associations with depression, anxiety, and clinical characteristics,” by Simsekoglu et al. 1 offers valuable insight into the psychological dimensions of migraine and highlights the potential relevance of self-compassion (SC) in understanding emotional distress among individuals with migraine. The study is commendable for examining an underexplored area and for evaluating SC across clinically-relevant migraine subgroups. However, several methodological and statistical considerations may warrant further clarification to strengthen interpretation of the findings.

One important consideration relates to subgroup sample sizes. Although the overall migraine cohort included 98 participants, only 9 individuals were classified as having chronic migraine and 10 as having medication-overuse headache. Given that multiple comparisons were conducted across SC subdomains as well as anxiety and depression measures, the limited size of these subgroups may reduce statistical precision and increase uncertainty surrounding subgroup-specific estimates. Consequently, findings involving these smaller clinical categories should be interpreted cautiously. 2

Another issue concerns psychiatric assessment procedures. Participants with major psychiatric disorders were excluded using clinical interview and medical record review; however, no structured diagnostic instrument was reported. Given the well-established overlap between migraine, anxiety, and depressive symptoms, the absence of standardized psychiatric assessment may increase susceptibility to residual confounding or diagnostic misclassification. 3 Incorporating validated structured interviews would improve methodological consistency and reproducibility.

The statistical analysis also raises several concerns. A large number of hypothesis tests were conducted across SC subdomains, migraine characteristics, psychological scales, and correlation analyses. Although Bonferroni adjustment was mentioned in one subgroup table, the manuscript does not clearly indicate whether correction for multiple testing was consistently applied throughout all analyses. In studies involving numerous simultaneous comparisons, incomplete adjustment may increase the probability of type-I error. 4 Clearer reporting of multiplicity-control procedures would, therefore, strengthen confidence in the associations reported.

Furthermore, several migraine-related clinical variables, including migraine duration, aura status, medication overuse, and pain severity, demonstrated associations with SC in correlation analyses but were not incorporated into the final regression model. Exclusion of these clinically-relevant factors may leave residual confounding factors unaddressed and limit interpretation of the independent contribution of psychological variables. 5

An additional limitation concerns the cross-sectional nature of the study. Although lower self-compassion was associated with greater depressive and anxiety symptoms, the temporal direction of these relationships cannot be determined. It remains unclear whether reduced self-compassion contributes to psychological distress among individuals with migraine or whether depression and anxiety themselves negatively influence self-compassion scores. Consequently, reverse causality cannot be excluded, and longitudinal investigations will be necessary to clarify the directionality of these associations. 6

Finally, while several associations achieved statistical significance, greater emphasis on effect sizes and the clinical relevance of the observed relationships would facilitate interpretation of their practical importance. Statistical significance alone may not fully reflect the magnitude or clinical utility of the findings reported, particularly in studies involving multiple comparisons and heterogeneous clinical characteristics. 7

Overall, the study in question contributes meaningful preliminary evidence regarding the relationship between self-compassion and migraine-related psychological distress. Addressing these methodological considerations would further strengthen interpretation of the findings and guide future research in this emerging area.
